# Depression and anxiety among pregnant women living with HIV in Kilimanjaro region, Tanzania

**DOI:** 10.1371/journal.pone.0224515

**Published:** 2019-10-31

**Authors:** James Samwel Ngocho, Melissa H. Watt, Linda Minja, Brandon A. Knettel, Blandina T. Mmbaga, Petal P. Williams, Katherine Sorsdahl

**Affiliations:** 1 Kilimanjaro Christian Medical University College, Moshi, Tanzania; 2 Alan J Flisher Centre for Public Mental Health University of Cape Town, Cape Town, South Africa; 3 Duke Global Health Institute, Duke University, Durham, North Carolina, United States of America; 4 Kilimanjaro Clinical Research Institute, Moshi, Tanzania; 5 Alcohol, Tobacco and Other Drug Research Unit, South African Medical Research Council, Cape Town, South Africa; Yeshiva University Albert Einstein College of Medicine, UNITED STATES

## Abstract

**Introduction:**

Mental health disorders in pregnant women living with HIV are associated with poor maternal and child outcomes, and undermine the global goals of prevention of mother-to-child transmission of HIV (PMTCT). This study aimed to determine prevalence of depression and anxiety and identify factors associated with these common mental health disorders among HIV-infeced pregnant women in Tanzania.

**Methods:**

We enrolled 200 pregnant women living with HIV from antenatal care clinics in the Kilimanjaro region. Women were eligible if they were in the second or third trimester of pregnancy and had been in PMTCT care for a minimum of one month. Data were collected via interviewer administered surveys. Participants self reported depression symptoms (Edinburgh Postnatal Depression Scale, EPDS) and anxiety symptoms (Brief Symptom Index, BSI). Multivariate logistic regression models examined factors associated with depression, anxiety, and comorbid depression and anxiety.

**Results:**

25.0% of women met screening criteria for depression (EPDS ≥10). Depression was significantly associated with being single (aOR = 4.2, 95% CI = 1.1–15.5), food insecurity (aOR = 2.4, 95% CI = 1.0–6.4), and HIV shame (aOR = 1.2, 95% CI = 1.1–1.3). 23.5% of participants met screening criteria for anxiety (BSI ≥1.01). Anxiety was associated with being single (aOR = 3.6, 95%CI = 1.1–11.1), HIV shame (aOR = 1.1, 95% CI = 1.1–1.2) and lifetime experience of violence (aOR = 2.3, 95% CI = 1.0–5.1). 17.8% of the sample met screening criteria for both depression and anxiety. Comorbid depression and anxiety was associated with being single (aOR = 4.5, 95%CI = 1.0–19.1), HIV shame (aOR = 1.2, 95%CI = 1.1–1.3) and lifetime experience of violence (aOR = 3.4, 95% CI = 1.2–9.6).

**Conclusion:**

Depression and anxiety symptomatology was common in this sample of pregnant women living with HIV, with a sizable number screening positive for comorbid depression and anxiety. In order to successfully engage women in PMTCT care and support their well-being, strategies to screen for mental health disorders and support women with mental illnesses are needed.

## Background

The HIV epidemic remains a significant global health challenge. At the end of 2018, an estimated 36.9 million people were living with HIV worldwide, with the large majority being in sub-Saharan Africa [1]. In Tanzania, an estimated 1.5 million people are living with HIV, with substantially higher prevalence among women (6.2%) compared to men (3.8%) [2]. Although HIV affects people of all ages, women of childbearing age are at a greater risk of acquiring HIV compared with their counterpart. In 2011, the prevalence of HIV among women attending antenatal clinics in Tanzania was reported at 5.6% [3].

Not only are people living with HIV (PLHIV) more likely to have a number of physical co-morbidities [4], but they are also more likely to suffer from common mental health disorders (CMDs) such as depression [5] and anxiety [6]. A meta-analysis of ten studies found depression to be nearly twice as high in PLHIV compared to the general population [7]. The current evidence supports the association between HIV infection and mental illnesses as a vicious cycle, where each illness is a risk factor for the other [8].

Pregnancy and the postpartum period are vulnerable times for depressive [9] and anxiety disorders [10]. Studies from low and middle income countries (LMICs) have reported a higher prevalence of depression and anxiety among pregnant women [11]. Further, in a systematic review of studies among HIV-positive African women, the prevalence of antenatal depression ranged between 23% and 44%, more than double the estimated rates in the general population of pregnant women [12].

Antenatal depression and anxiety are associated with a number of poor HIV-related maternal outcomes and child. For example, pregnant women with mental illness are at greater risk for poor adherence to antiretroviral therapy [13], which can lead to vertical transmission of HIV to the fetus and breastfeeding child [14], with up to 11% vertical transmission in Tanzania. Pregnant women living with HIV with depression progress faster to the advanced stages of HIV compared to those without depression [15], and they are two times more likely to die from AIDS-related deaths, especially in LMICs [15,16]. Additionally, there is evidence that antenatal anxiety among women living with HIV may negatively impact child growth and development [17].

A number of factors contribute to perinatal mental health disorders among pregnant women living with HIV, ranging from individual to health system level factors. A systematic review of studies in Ethiopia reported individual and inter-personal factors that are associated with perinatal depression. In their review younger age, unmarried women, low monthly income, employed, history of the previous mental health disorder, irregular or no prevous antenatal follow-up, unplanned pregnancy, previous maternal and/or fetal complication (s) during pregnancy, conflict, and low social support were associated with antenatal depression [18].

Mental health disorders in HIV-positive pregnant women must be understood in the context of women’s life circumstances. Many women are newly diagnosed with HIV during pregnancy. Receiving a positive HIV test result is a shocking life event, creating worry about one’s future and the fear of transmitting the virus to one’s unborn child [19]. Additionally, stigma and discrimination following HIV status disclosure remains of concern in many communities. In one study in Tanzania, 32% of women reported being discriminated against due to HIV, and 12% reported abandonment or divorce following their HIV status disclosure [20].

In Tanzania, although one study has investigated the prevalence of depression among pregnant women living with HIV [21], no studies have included anxiety disorder. Therefore, the present study determined the prevalence of both depression and anxiety among HIV-positive pregnant women in northern Tanzania and examined factors associated with these common depression and anxiety.

## Materials and methods

### Study design and setting

This was a cross-sectional study among HIV-positive pregnant women in the Kilimanjaro Region. Participants were recruited at nine antenatal clinics, including six from the Moshi municipality and three from the Moshi district.

### Participants

As per Tanzanian national PMTCT guidelines, all pregnant women who test positive for HIV initiate antiretroviral therapy (ART) for lifetime use [22]. Women were eligible to enroll in the study if they were in the second or third trimester of pregnancy and had enrolled in PMTCT care at one of the study antenatal clinics at least one month prior. Additionally, age 18 years and above and provided a written informed consent.

### Procedure

**Fig 1:**
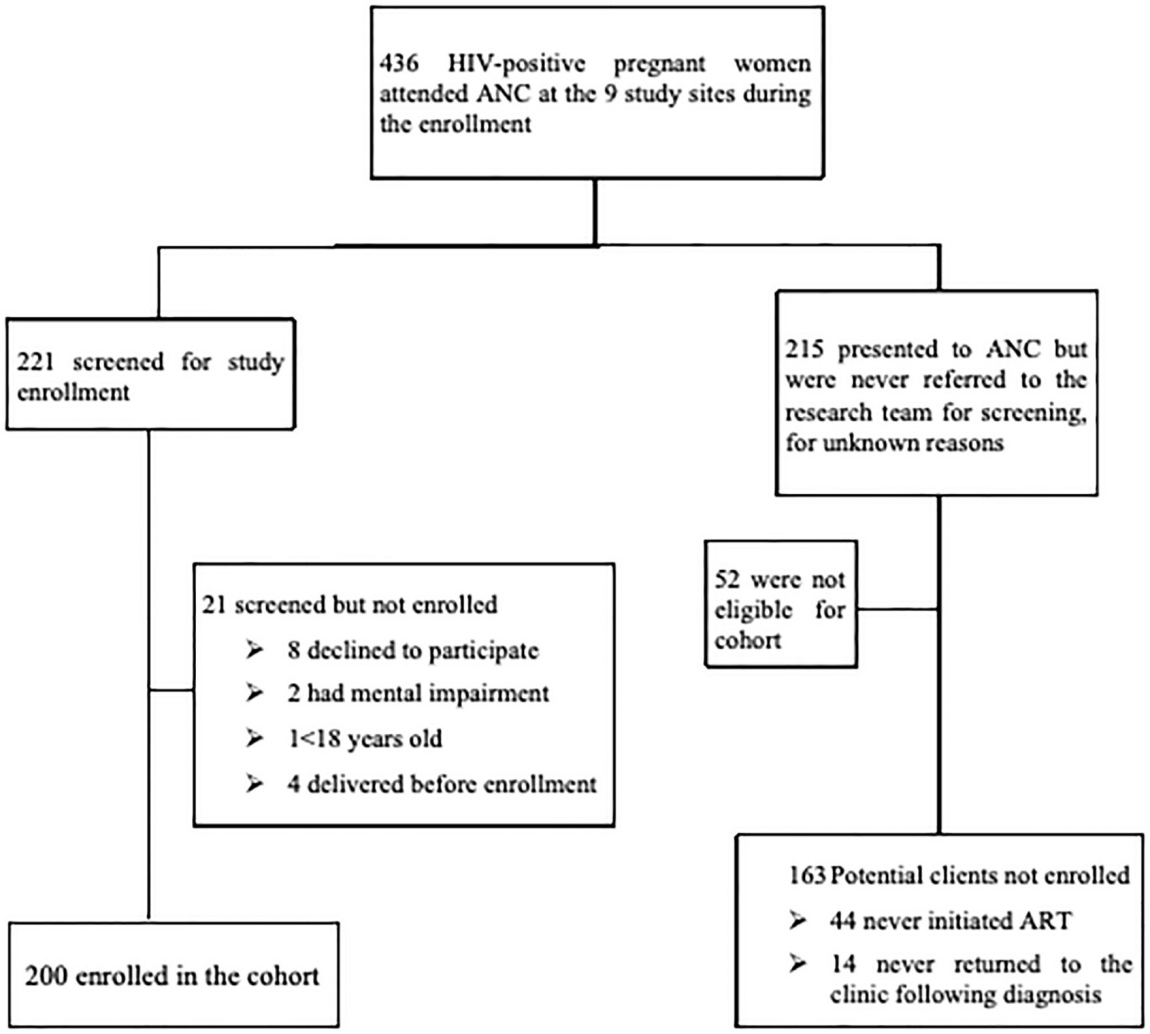
Participant screening and enrollment flow diagram.

The structured survey was based on the baseline survey of a 12 month longitudinal survey of HIV-positve pregnant women in the Kilimanjaro Region [23]. Pregnant women living with HIV presenting for their routine antenatal care were consecutively approached by the clinic nurses informed of the study and asked if they were interested in obtaining more information. Between Juy 2016 and August 2017 436 pregant women living with HIV who attended ANC were approached, 221 experessed interest in the study and were referred to the research office for screening and enrollment (Fig 1).

After obtaining informed consent, a structured survey was verbally administered in Swahili by a trained data collector. Study activities were conducted in private offices located within the study clinics. The assessment took approximately 60 minutes to complete. Participants were reimbursed for transportation costs (5,000 Tanzania shillings; approximately $2.30 U.S.) and were provided with light snacks during the interview. Participants with distress and thought of self-harm were counselled by the study nurse and also helped to create an individualized safety plan. Additionally, they were referred back to the clinic counsellor for further support.

### Measures

The data collection tools were translated into Swahili and then back-translated into English by two independent translators. If there was a difference between the two after back translation, a team of Swahili and English-speaking study staff reached consensus on the final wording.

In addition to socio-demographic variables (age, level of education, employment status, prior pregnancy, and relationship status), the following measures were included in the survey.

#### Depression

Depression was measured using the Edinburgh Postnatal Depression Scale (EPDS) [24]. The EPDS contains ten questions asking about depressive symptoms over the past seven days. Each question has four possible responses, with a score of 0 to 3. Items were summed, with a possible range of 0 to 30 and higher scores indicating more depression symptoms (α = 0.88). A score of 10 was used as a cut-off to indicate possible depression [25].

#### Anxiety

Anxiety was measured using the six-item anxiety subscale of the Brief Symptom Index (BSI-18) [26]. Items asked about anxiety symptoms over the past seven days, with response options on a 5-point scale, ranging from 0 (not at all) to 4 (extremely). Items were averaged, with a possible range of 0 to 5 (α = 0.92). Based on instrument norms for a non-clinical, female population, a score of 1.01 or higher was used as a cut-off to indicate probable anxiety [27].

#### Attitudes about pregnancy

An 8-item measure was adapted from Speizer and colleagues to assess attitudes about pregnancy [28]. Items were summed, with a possible range from 0 to 24 and higher scores indicating more positive attitudes about the pregnancy (α = 0.91).

#### Intimate partner violence

The modified WHO intimate partner violence tool was used to assess for history of intimate partner violence, including questions about emotional, physical, and sexual abuse. The violence scores were dichotomised, with a yes to any of the aforementioned questions being indicative of a lifetime history of violence [29].

#### Enacted Stigma

A stigma measure was adapted from the Holzemer HIV/AIDS Stigma Instrument (HASI) [30]. The 11 items asked about stigmatizing experiences as a result of HIV (e.g., someone stopped being a friend). Items were summed, with a possible range from 0 to 33 and higher scores indicating greater experience with stigma (α = 0.88).

#### HIV Shame

The HIV and Abuse Related Shame Inventory (HARSI) was used to measure HIV shame [31]. The current study adapted 13 statements from the 14-item HIV-related shame subscale of the measure. The 13 items asked about internalized feelings related to living with HIV (e.g., I put myself down for becoming HIV positive, I am ashamed that I’m HIV positive). Items were summed, with a possible range from 0 to 52 and higher scores indicating greater shame (α = 0.86).

#### Food security

Four items from the Household Food Insecurity Access Scale (HFIAS) were used to assess household food availability over the past 30 days [32]. We adapted four questions from the nine-item household food insecurity scale (e.g. In the past month, how often could you not feed your family?). The measure was dichotomized into whether or not someone reported any food insecurity.

#### Social support

The Perceived Availability of Support Scale (PASS) was used to measure social support [33]. The participants were asked to respond to 8 questions (e.g., Would someone be available to talk to you if you were upset, nervous, or depressed?). Scores ranged from 8 to 40 with higher scores indicating greater perceived support from others (α = 0.82).

Of the tools used to measure outcomes and the explanotory variables, only food security and enacted stigma assessment tools have been validated in Tanzania [32]. Though not in Tanzania, some have been validated in other East African countries, for example, anxiety measure (BSI) has been validated in Kenya [34]

#### HIV-related variables

Women enrolled were asked if they had disclosed their HIV status to anyone or a sexual partner (Have you told anyone about your HIV status?). Also, their partner HIV status.

### Ethical consideration

Ethical approval for the study was provided by the University of Cape Town Human Research Ethics Committee. The ethical review boards of the Tanzanian National Institute for Medical Research and Kilimanjaro Christian Medical University.

Eligible participants were asked to sign a form providing informed consent before their participation. Participants who could not read or write were asked to provide a thumbprint and their consent was verified by the signature of an impartial witness of the participant’s choice.

### Data analysis

Stata Version 14.0 was used to analyse the data. Frequency distributions and descriptive statistics were calculated for categorical and continuous variables. Three multivariate logistic regression models were developed to assess the factors associated with depression, anxiety, and comorbid depression and anxiety. To control for confounders and reduce residual confounding effects, factors with a p-value of 0.15 or less in bivariate analysis were considered eligible for inclusion in the multivariate analysis, along with demographic variables (age, level of education and marital status) that were selected *a priori*. Factors with a p-value of less than 0.05 in the final model were considered statistically significant.

## Results

A total of 208 pregnant women living with HIV were referred and deemed eligible for participation. Of those, 8 (3.8%) declined to participate, leaving a final sample of 200. The median age was 30 years (IQR 25–35). About half of participants knew they were HIV-positive prior to the current pregnancy (n = 106, 53.0%), and 88 (44.0%) had initiated ARV prior to the current pregnancy. When asked whether they had disclosed their status to anyone, 159 (79.5%) had disclosed to at least one person, and more than two-thirds (n = 119, 70.0%) reported that they had disclosed to a male partner (Table 1).

**Table 1:** Characteristics of study participants (n = 200).

Characteristics	n	%
*Median(IQR) age in years*	30 (25–35)	
*Education level attained*		
No formal education	4	2.0
Any primary education	113	56.5
Any secondary education	70	35.0
Any college	13	6.5
*Marital status*		
Married	98	49.0
In a relationship	74	37.0
Single	19	9.5
Separated/Divorced	9	4.5
*Polygamous relationship*		
Yes	36	18.0
No	164	82.0
*Income-earning activities*		
None	60	30.0
Informal activities	111	55.5
Formal employment	29	14.5
*First pregnancy*		
Yes	44	22.0
No	156	78.0
*Gestational age mean (±SD)*	28.4 (±5.6)	
*Prior pregnancy outcome (n = 156)*		
No problems	85	54.5
Negative outcomes	71	45.5
*Negative outcomes (n = 71)*		
Abortion	7	9.9
Miscarriage	32	45.1
Still birth	12	16.9
HIV+ child	11	15.5
Death of a child	23	32.4
*HIV diagnosis*		
New diagnosis	94	47.0
Established diagnosis	106	53.0
*Any HIV status disclosure*		
Yes	159	79.5
No	41	20.5
*Disclosed to partner (n = 172)*		
No	53	16.2
Yes	119	83.8

Fifty (25.0%) women met screening criteria for possible depression. Twenty-eight (14.1%) participants reported thoughts of self-harm, of whom 8 reported that they had these thoughts “quite often”. Forty-nine (24.6%) met screening criteria for probable anxiety disorder. The prevalence of comorbid anxiety and depression was 18.1% (n = 36). The majority (72%) of women who met screening criteria for depression also met screening criteria for anxiety.

In the first multivariate logistic regression model examining factors associated with depressive symptoms (Table 2), relationship status, food insecurity and HIV shame were significantly associated with depression. Women who were single had 4.2 times higher odds of possible depression compared with women who were married or in a relationship (aOR = 4.2, 95% CI = 1.1–15.5). Women who reported some food insecurity had 2.4 times higher odds of possible depression compared with those without food insecurity (aOR = 2.4, 95% CI = 1.0–6.4). Women with greater HIV shame had higher odds of depression; with each unit increase in HIV shame score, the odds of depression increased by 1.2 (aOR = 1.2, 95% CI = 1.1–1.3).

**Table 2:** Factors associated with depression among pregnant women living with HIV (n = 200).

Variables	Possible depression		cOR (95%CI)	p-value	aOR (95%CI)	p-value
	No (n = 150) n(%)	Yes (n = 50) n(%)				
*Education level attained*						
Primary	83(70.9)	34 (29.1)	1.7(0.9–3.4)	0.118	1.6(0.6–4.1)	0.314
Secondary and above	67 (80.7)	16 (19.3)	Ref			
*Marital status*						
Married	135 (78.5)	37 (21.5)	Ref		Ref	
Single	15 (53.6)	13 (46.4)	3.2(1.4–7.2)	0.006	4.2 (1.1–14.6)	0.030
*Income-earning activities*						
None	43 (71.7)	17 (23.8)	1.2(0.6–2.5)	0.477		
Informal/Formal	107 (76.4)	33 (23.6)	Ref			
*First pregnancy*						
Yes	34 (77.3)	10 (22.7)				
No	116 (74.4)	40 (25.6)	1.2(0.5–2.6)	0.694		
*HIV diagnosis*						
New diagnosis	71 (75.5)	23 (24.5)	Ref			
Established diagnosis	79 (74.5)	27 (25.5)	1.0(0.5–2.0)	0.870		
*Disclosure to anyone*						
No	34 (82.9)	7 (17.1)	0.5 (0.2–1.3)	0.193		
Yes	116 (73.0)	43 (27.0)				
Yes	91 (76.5)	28 (23.5)				
*Food Insecurity (FHI)*						
Never	112 (84.2)	21 (15.8)				
Sometimes	38 (56.7)	29 (43.3)	4.1 (2.1–8.0)	<0.001	2.4 (1.0–6.4)	0.056
*Ever experienced violence*						
No	106 (81.5)	24 (18.5)				
Yes	44 (62.9)	26 (37.1)	2.6 (1.3–5.0)	0.004	1.5 (0.6–3.6)	0.409
Variables	Median(IQR)	Median(IQR)	cOR (95%CI)	P value	aOR	P-value
Age in years	30 (26–35)	30 (24–35)	1.0 (0.9–1.0)	0.532	1.0 (0.9–1.1)	0.892
Social support	30 (24–36)	26.5 (23–30)	0.9 (0.9–1.0)	0.001	1.0 (0.9–1.0)	0.303
HIV Shame	15 (10.7–20)	29 (22.7–35)	1.2 (1.1–1.3)	<0.001	1.2 (1.1–1.3)	<0.001
Enacted stigma	0 (0–0)	0 (0–2)	1.3 (1.0–1.5)	0.014	1.0 (0.9–1.2)	0.621
Attitude about pregnancy	16 (13–19.5)	14 (9.7–16)	0.9 (0.9–1.0)	0.007	0.9 (0.8–1.0)	0.134

cOR, crude odds ratio, aOR, adjusted odds ratio

Adjusted for social support, enacted stigma, attitude about pregnancy, HIV shame, level of education, marital status, food insecurity and violence

In the second multivariate logistic regression model examining factors associated with probable anxiety (Table 3), relationship status, HIV shame and lifetime experience of violence were significantly associated with anxiety. Women who were single had 3.6 times higher odds of probable anxiety compared with women who were married or in a relationship (aOR = 3.6, 95% CI = 1.1–11.1). Women with greater HIV shame had higher odds of probable anxiety; a one unit increase in HIV shame score was associated with 1.1 times increased odds of probable anxiety (aOR = 1.1, 95% CI = 1.1–1.2). Women who reported a history of lifetime violence had 2.3 times higher odds of probable anxiety compared to those who had never experienced violence (aOR = 2.3, 95% CI = 1.0–5.1).

**Table 3:** Bivariate and multivariate analysis of factors associated with probable anxiety among pregnant women living with HIV (n = 199).

Variables	Probable anxiety		cOR (95%CI)	p-value	aOR(95%CI)	p-value
	No(n = 150) n(%)	Yes(n = 49) n(%)				
*Education level attained*						
Primary/No formal	84 (71.8)	33 (28.2)	1.6 (0.8–3.2)	0.163	1.5(0.6–3.4)	0.363
Secondary and above	66 (80.5)	16 (19.5)				
*Marital status*						
Married	135 (78.9)	36 (21.0)				
Single	15 (53.6)	13 (46.4)	3.2 (1.4–7.4)	0.005	3.6 (1.1–11.1)	0.027
*Income-earning activities*						
None	43 (71.7)	17 (28.3)	1.3 (0.7–2.6)	0.426		
Informal/Formal	107 (77.0)	32 (23.0)				
*First pregnancy*						
Yes	30 (69.8)	13 (30.2)	1.4 (0.7–3.0)	0.336		
No	120 (76.9)	36 (23.1)				
*HIV diagnosis*						
New diagnosis	71 (76.3)	22 (23.7)				
Established diagnosis	79 (74.5)	27 (25.5)	1.1 (0.6–2.1)	0.767		
*Partner recent test*						
HIV-negative/Unknown	101 (73.2)	37 (26.8)	1.5 (0.7–3.1)	0.283		
HIV-infected	49 (80.3)	12 (19.7)				
*Disclosure to anyone*						
No	32 (78.0)	9 (22.0)				
Yes	118(74.7)	40 (25.3)	1.2 (0.5–2.7)	0.748		
*Food Insecurity (FHI)*						
Never	107 (81.1)	25 (18.9)				
Sometimes	43 (64.2)	24 (35.8)	2.4 (1.2–4.6)	0.010	1.2 (0.5–2.8)	0.676
*Ever experienced violence*						
No	107 (82.9)	22 (17.1)				
Yes	43 (61.4)	27 (38.6)	3.0 (1.6–5.9)	0.001	2.3 (1.0–5.1)	0.045
Variables	Median(IQR)	Median(IQR)	cOR(95%CI)	p-value	aOR(95%CI)	p-value
*Age in years*	30.5 (26–35)	28 (24–35)	1.0 (0.9–1.0)	0.153	1.0 (0.9–1.0)	0.445
*Social support(PAS)*	29(24–34)	27 (23–31.5)	1.0 (0.9–1.0)	0.104	1.0 (1.0–1.1)	0.566
*HIV Shame*	15.5(11–21.2)	28 (17–34.5)	1.1 (1.1–1.2)	<0.001	1.1 (1.1–1.2)	<0.001
*Enacted stigma*	0 (0–0)	0 (0–2)	1.3 (1.1–1.6)	0.011	1.1 (0.9–1.3)	0.205
*Attitude about pregnancy*	16 (12.4–19)	14(9.5–19)	0.9 (0.9–1.0)	0.081	0.9 (0.9–1.0)	0.351

crude odds ratio, cOR; adjusted odds ratio, aOR

adjusted for level of education, marital status, perceived availability of social support, HIV shame, enacted stigma and attitude about current pregnancy

One participant did not complete anxiety screening instrument (BSI)

In the third multivariate logistic regression model examining factors associated with comorbid symptoms of depression and anxiety (Table 4), relationship status, HIV shame and lifetime experience of violence were significant predictors. Women who were single had 4.5 times higher odds of possible comorbid depression and anxiety compared with women who were married or in a relationship (aOR = 4.5, 95% CI = 1.0–19.1). Women with greater HIV shame had higher odds of experiencing comorbid depression and anxiety; for each unit increase in HIV shame score, the odds of comorbidity increased by 1.2 times (aOR = 1.2, 95% CI = 1.1–1.3). Women who had a lifetime history of violence had 3.5 times higher odds of comorbid depression and anxiety, compared to those with no history of violence (aOR = 3.5, 95% CI = 1.3–9.9).

**Table 4:** Bivariate and multivariate analysis of factors associated with comorbidity of depression and anxiety (n = 199).

Variables	Probable Anxiety and possible Depression		cOR(95%CI)	p-value	aOR(95%CI)	p-value
	No (n = 163) n(%)	Yes (n = 36) n(%)				
Education level attained						
Primary/No formal	92 (78.6)	25 (21.4)	1.7 (0.8–3.8)	0.155	1.6(0.5–4.8)	0.379
Secondary/Higher education	73 (86.6)	11 (13.4)				
*Marital status*						
Married	146 (85.4)	25 (14.6)				
Single	17 (60.7)	11 (39.3)	3.8 (1.6–9.0)	0.003	4.5 (1.0–19.1)	0.042
*Income-earning activities*						
None	46 (76.7)	14 (23.3)	1.6 (0.8–3.4)	0.209		
Informal/Formal	117 (84.2)	22 (15.8)				
*First pregnancy*						
No	129 (82.7)	27 (17.3)				
Yes	34 (79.1)	9 (20.9)	1.3 (0.5–2.9)	0.585		
*HIV diagnosis*						
New diagnosis	78 (83.9)	15 (16.1)				
Established diagnosis	85 (80.2)	21 (19.8)	1.3 (0.6–2.7)	0.501		
*Partner recent test*						
HIV-negative/Unknown	110 (79.7)	28 (20.3)	1.7 (0.7–3.9)	0.229		
HIV-infected	53 (86.9)	8 (13.1)				
*Disclosure to anyone*						
No	36 (87.8)	5 (12.2)	0.6 (0.2–1.6)	0.276		
Yes	127 (80.4)	31 (19.6)				
*Food Insecurity (FHI)*						
Never	117 (88.6)	15 (11.4)				
Sometimes	46 (68.7)	21 (31.3)	3.6 (1.7–7.5)	0.001	1.5(0.5–4.5)	0.420
*Ever experienced violence*						
No	116 (89.9)	13 (10.1)				
Yes	47 (67.1)	23 (32.9)	4.4 (2.1–9.3)	<0.001	3.5(1.3–9.9)	0.016
Variables	Median(IQR)	Median(IQR)	cOR(95%CI)	p-value	aOR(95%CI)	p-value
Age	30 (26–35)	28 (23–35)	0.9 (0.9–1.0)		1.0 (0.9–1.1)	0.575
*Social support(PAS)*	30 (24–35)	26 (22.2–29)	0.9 (0.9–1.0)	0.004	1.0 (0.9–1.1)	0.515
*HIV Shame (HARSIH)*	16 (11–22)	30 (23–36)	1.1 (1.1–1.2)	<0.001	1.2 (1.1–1.3)	<0.001
*Enacted stigma(HASI)*	0 (0–0)	0 (0–3)	1.3 (1.1–1.6)	0.006	1.1 (0.9–1.3)	0.186
*Attitude about pregnancy(PREGAT)*	16 (12.6–19)	14 (9–16.7)	0.9 (0.8–1.0)	0.010	0.9 (0.8–1.0)	0.150

cOR, crude odds ratio; aOR, adjusted odds ratio, CI, confidence interval

adjusted for marital status, violence, food insecurity, attitude about current pregnancy, enacted stigma, perceived availability of social support, and HIV shame

## Discussion

The main results of this study are that: 1) the prevalence of possible depression was 25% and was associated with relationship status, food insecurity, and HIV shame; 2) symptoms of anxiety were common (24.6%) and were associated with relationship status, HIV shame and lifetime experience of violence; and 3) a high rate of comorbidity was reported by participants, with 18.1% meeting criteria for both possible depression and probable anxiety. HIV shame, relationship status, and lifetime experience of violence were found to be significantly associated with comorbid depression and anxiety.

In the present study, one out of every four pregnant women living with HIV had possible depression. Although these findings are comparable to the US prevalence of 22% [35], compared to studies conducted in other African countries and other studies in Tanzania, this prevalence is low. For example, in Uganda and South Africa, depression rates of 42.7% and 41.0% have been reported, respectively [36,37]. Similarly, in Dar es Salaam, the prevalence of antenatal depression among women living with HIV was reported to be about twice (42.4%) or three times (74.3%) that of the prevalence in Moshi [38,39]. There are a number of explanations that may account for the different rates of depression reported in the present study compared to the available literature in other African countries. The use of different depression screening tools might have contributed to the observed difference in the prevalence. In the previous studies conducted in Tanzania and Uganda, the Hopkins Symptoms Checklist [40] was used to screen for depression [39], while this study used EPDS [36]. Also, the difference in the study design of the previous studies might have contributed to the observed difference. For instance, in the mentioned study in Tanzania, participants were enrolled from an ongoing trial [39], while the current study is cross-sectional.

Similar to prevalence rates of depression reported in the present study, 24.6% of women screened positive for anxiety. One study in the US reported a much higher prevalence (71.1%) compared to what we found [10]. However, that study had a sample size of 45. The sample size, study design, anxiety measures, and the difference in the cultural context of mental illness might have contributed to the observed difference. In Sub-saharan Africa, there is a paucity of data on the prevalence of anxiety among pregnant women living with HIV.

Interestingly, in the present study, 18.1% of women met screening criteria for comorbid depression and anxiety. Literature shows that the comorbidity of anxiety and depression is common among pregnant women, similar to the general population (10%) [41]. However, the data in pregnant women living with HIV is limited. Previous literature suggests that comorbidity is even more common compared to the presence of either depression or anxiety alone, with up to 52% of low income pregnant women in one study in urban South Africa diagnosed with depression also presenting symptoms of anxiety [42].

A number of factors were associated with these CMDs among pregnant living with HIV. To begin with, single women were at higher risk of depression, anxiety and comorbid compared to married women. The results are consistent with one previous study in the USA [10]. Being single and pregnant might not be a women’s choice, but rather may be a result of unintended pregnancies or being abandoned by a partner for reasons related to pregnancy, HIV or both. The circumstances resulting in a pregnant woman being single in the Tanzanian context might contribute to depression.

Second, this study found that women who reported food insecurity in their households had higher odds of CMDs. Associations between food insecurity and depression have been highlighted in Uganda, South Africa and the United States [43–45]. In addition to the stress of not having sufficient food for one’s household, there is an added stress of not being able to take the advice of HIV health care providers to eat a balanced diet and take HIV medication with food. The finding raises concerns about social disparities among PLHIV and shows that interventions should not only focus on physical health but should also address the socio-economic circumstance of clients.

Third, pregnant women who had greater HIV shame had higher odds of anxiety. One study from Bangkok Thailand reported similar findings [46]. Many PLHIV feel ashamed of their status and feel that they have let their families down [47]. Additionally, many believe that having HIV is a punishment for their behaviour [47,48], resulting in self blame and distress. One strategy that individuals use to cope with a shameful life event is to avoid the related events and situations that bring back shameful memories [47], in this case, avoidance is not an option adding to the risk of anxiety. Women who are in HIV care and take medications have a constant reminder of their HIV status, which may contribute to anxiety. Despite the fact that HIV is now a treatable chronic condition, it remains a stigmatized condition. In particular, since HIV transmission is commonly associated with sexuality, women in particularly may feel that they have diverged from the norm and feel ashamed as a result putting them at risk of anxiety [49].

Finally, women with a history of violence were at increased risk of anxiety and co-morbid anxiety and depression. Because women often learn their status first in a relationship through routine antenatal HIV testing [22], they may be blamed for introducing the disease into the relationship [50]. Because of this, some marriages end in separation or divorce, and others may be the victims of violence [20]. In order to achieve the ambitious targets set forth, more efforts are needed to overcome HIV-related shame, violence, and resulting symptoms of mental health disorders.

This study has demonstrated that mental health disorders are common in pregnant women living with HIV in the two districts of the Kilimanjaro region Tanzania. Although both depression and anxiety are common in this population, mental health services are nearly non-existent and there are no clear strategies or guidelines for medical staff on how to screen, diagnose and manage these conditions. Antenatal care facilities, including PMTCT clinics, are a vital entry point to detect and manage depression and anxiety among this vulnerable population. The findings of this study point to the need to train and deploy mental health care providers in order to provide mental health services at the PMTCT clinics. In order to successfully engage women in HIV care and support their well-being, and in turn eliminate mother to child transmission of HIV, strategies to screen for mental health and support women with mental health disorders are needed.

### Limitations

The results of this study should be interpreted with some caution. The study enrolled only the subset of women attending the antenatal clinic who were referred to the study, and those who were not recruited may have differed in their presentation. Some of the assessment tools have not been validated in Tanzania, which might have impacted our findings, and cut off scores for depression and anxiety were based on non-Tanzanian norms. Finally, the cross sectional design of this study precludes any conclusions about causation.

### Conclusion

In this population of women living with HIV, we found a substantial prevalence of possible depression, anxiety, and comorbid depression and anxiety. In order to meet the target of elimination of mother-to-child transmission of HIV, mental health needs to be addressed with a similar emphasis to that of physical health. Unmarried women, women with a history of experiencing violence, women with food insecurity, and those who felt ashamed (i.e., self-stigma) related to their HIV had higher odds of mental illness. These factors are deeply rooted in culture and personal beliefs, which emphasies the importance of tailored interventions that provide psycho-social support that reflect the local context.

## Supporting information

S1 Dataset (XLS)
